# Toxoplasmosis in pregnancy; analysis of maternal seropositivity in a large cohort in Turkey and clinical consequences of neonates

**DOI:** 10.1097/MD.0000000000044881

**Published:** 2025-10-03

**Authors:** Bilge Cetinkaya Demir, Oguzhan Yuruk, Yasemin Heper, Hilal Ozkan

**Affiliations:** a Department of Obstetrics and Gynecology, Faculty of Medicine, Bursa Uludag University, Bursa, Turkey; b Department of Infectious Disease, Faculty of Medicine, Bursa Uludag University, Bursa, Turkey; c Department of Neonatology, Faculty of Medicine, Bursa Uludag University, Bursa, Turkey.

**Keywords:** congenital toxoplasmosis, IgG avidity, seropositivity, *Toxoplasma gondii*

## Abstract

Toxoplasmosis infestation during pregnancy is typically asymptomatic however, the risk of vertical transmission increases with advancing gestational age. Due to the absence of overt clinical symptoms in most pregnant women, diagnosis relies on serological testing. Seroprevalence rates vary widely depending on geographical location and cultural practices. In the fetus, toxoplasmosis can result in a spectrum of outcomes, ranging from asymptomatic infection to severe neurological impairment. This study aimed to determine the seroprevalence of *Toxoplasma gondii* during pregnancy and to evaluate fetal transmission in seropositive pregnant women. For those diagnosed with acute toxoplasmosis, neonatal examination, serological tests, and transfontanel ultrasonography were conducted. This retrospective cohort study evaluated Anti-Toxoplasma IgM, IgG, and IgG avidity results of patients who presented to a tertiary medical center between 2015 and 2020. All maternal serological results, ultrasonographic evaluations, and neonatal data (e.g., serology, transfontanel ultrasonography) from births in the institution were obtained from hospital records. During the study period, 69 out of 2137 women (3.2%) had positive or borderline Toxoplasma IgM results. Seventeen patients were considered to have preconceptional infection, other 46 were suspected as acute toxoplasmosis during pregnancy. Three patients were lost to follow-up, and 5 pregnancies were terminated. Overall, 46 patients (2.1%) were started on spiramycin treatment by a mean gestational age of 14 weeks. Amniocentesis was performed in 14 cases, all of which had negative PCR results. Among neonates with available data, no findings suggestive of congenital toxoplasmosis were detected. The maternal seroprevalence of *Toxoplasma gondii* observed at our center aligns with findings from similar studies. 2 percent of 2137 women were suspected as acute toxoplasmosis and none of the 38 babies had neonatal intracranial abnormalities. However, the low acceptance rate (30%) for fetal diagnostic testing and the lack of complete neonatal serologic data may contribute to the observed low rate of congenital toxoplasmosis.

## 1. Introduction

*Toxoplasma gondii* infection is prevalent worldwide and is typically asymptomatic. However, it can cause severe disease in humans, particularly in congenitally infected children and immunocompromised individuals, low sanitary households and cases of fatal toxoplasmosis have also been reported in immunocompetent persons. Limited risk factor analysis data indicated that eating and handling raw meat, contact with contaminated soil and cat litter and consumption of raw eggs, were the main factors associated with seroprevalence.^[1]^

Congenital toxoplasmosis is a clinical condition that occurs in infants born to mothers who experienced acute infection 6 to 8 weeks before or during pregnancy. Its incidence is estimated to be between 0.1 and 0.3 per 1000 live births.^[1–3]^ Throughout pregnancy, the transmission rate to the fetus is approximately 30%, though the rates of transmission and fetal infection vary across trimesters.^[2,3]^ The rate of maternal seroconversion during pregnancy is reported to be 2 per 1000.^[4]^ While the risk of fetal transmission is lower in the early weeks of pregnancy (15% in the first trimester), the clinical outcomes are more severe. Based on data from Syrcot study, the incidence of neonatal cerebral and ocular lesions secondary to acute toxoplasmosis during pregnancy varies between 5% and 50%, contingent upon the geographical region, declining by gestational age.^[5]^

If treatment is initiated within 3 weeks following maternal seroconversion, the fetal transmission rate can be reduced by 52%.^[3,6]^ Approximately 20% to 25% of infants born with congenital toxoplasmosis display clinical symptoms.^[6]^ As the gestational age at the time of seroconversion increases, the incidence of intracranial findings decreases.^[6]^ Additionally, sequelae such as psychomotor or intellectual impairment, epilepsy, vision loss, and hearing loss may occur. Congenital toxoplasmosis can also result in stillbirth, preterm birth, or spontaneous abortion.^[1–3]^ When treatment is initiated after maternal infection, it can reduce both mortality and the incidence of severe neurological sequelae by 72%.^[6]^

Toxoplasma infection in pregnancy is usually asymptomatic and can only be detected by serological testing. Differentiating between acute and chronic toxoplasmosis infections is based on serological data and avidity testing.^[3]^ Anti-Toxoplasma IgM (IgM) can persist for months or even years after an acute infection, making it challenging to distinguish between acute and chronic infection based on this marker alone.^[3,6,7]^ Antenatal ultrasonography fails to detect abnormalities in one-third of affected fetuses.^[1,3,6]^ Toxoplasma gondii can be detected in amniotic fluid by PCR, which offers 100% specificity and a sensitivity range of 65% to 90%.^[8]^

Although the risk of congenital toxoplasmosis stems from acute toxoplasma infection during pregnancy, all national guidelines currently do not recommend routine toxoplasmosis screening during pregnancy.^[9,10]^ How often maternal infections are missed has not been assessed in countries where screening is not in routine. A few countries, including France and Austria, provide serological screening to pregnant women.

Currently, there is no nationally implemented congenital toxoplasmosis screening program in Turkey. However, at our institution, we routinely conduct serological screening for toxoplasmosis in pregnant women during their first-trimester visit. This study aims to assess the prevalence of Toxoplasma gondii IgM seropositivity in pregnant women attending a tertiary hospital, overview the diagnosis and treatment protocols for seropositive patients, and evaluate neonatal serology results, transfontanel ultrasonography, and clinical findings indicative of congenital toxoplasmosis in newborns.

## 2. Materials and methods

This retrospective cohort study evaluated *Toxoplasma gondii* seropositivity in pregnant women and neonatal serological outcomes of those who attended the Perinatology and Antenatal Clinic at a tertiary medical center, between January 2015 and January 2020. Pregnant women attending for follow-up in their first trimester are routinely checked for serologic tests for Toxoplasma gondii. Women who did not undergo Anti-Toxoplasma IgM (Toxo-IgM) and IgG (Toxo-IgG) testing during pregnancy were excluded from the study. In cases of serological positivity, IgM, IgG, and IgG avidity tests were repeated at 2-week intervals for confirmation. Spiramycin treatment was started at a dose of 1 g (3 million UI) orally every 8 hours for patients with a diagnosis of acute infection until confirmation with aminosynthesis.

Serological tests were conducted at the Microbiology ELISA Laboratory of Bursa Uludağ University Faculty of Medicine. Anti-Toxoplasma IgM and IgG antibodies were detected using VIDAS TOXO-IgG and IgM kits (bioMérieux, France), based on enzyme-linked fluorescent assay technology. IgG avidity was measured using VIDAS TOXO-IgG Avidity kits (bioMérieux, France), in accordance with the manufacturer’s instructions. Test results were interpreted in accordance with the manufacturer’s instructions as negative, borderline or positive for IgM and IgG. All kits were subject to routine internal and external quality control, and all results were documented. Initial serology tests were labeled as Toxo-IgM1/ IgG1/ Avidity1, and follow-up tests were labeled as Toxo-IgM2/ IgG2/ Avidity2.

Amniotic fluid PCR testing for *Toxoplasma* was recommended for pregnant women suspected of acute infection based on serologic results. Amniocentesis was routinely performed in these cases, either at 18 weeks of gestation or 6 weeks after the detection of acute infection.

Patient records were reviewed to obtain data on demographic characteristics and potential risk factors, including immunocompromised status, pet ownership, place of residence, and occupational exposure. Additional information extracted included the acceptance rate of amniocentesis, Toxoplasma PCR positivity in amniotic fluid, treatment modifications informed by test results, and fetal ultrasonographic findings from diagnosis to delivery. Neonatal serological results and transfontanelle ultrasonography findings were compared during postnatal follow-up for congenital toxoplasmosis. For Toxo-seropositive women who delivered at institutions other than our center, neonatal data were obtained through follow-up telephone interviews with parents and from nationwide neonatal surveillance system records.

The study protocol was approved by the Clinical Research Ethics Committee of Bursa Uludağ University Faculty of Medicine (approval number: 2020-2/6).

## 2.1. Outcome measure

The primary outcome of this study was the identification of Toxo-seropositive pregnant women and their infants who presented to the Perinatology and Antenatal Clinic. Secondary outcomes included the evaluation of neonatal *T. Gondii* serologic tests, transfontanelle ultrasonography findings and the presence of clinical anomalies.

## 2.2. Statistical analysis

The normality of continuous variables was assessed using the Shapiro–Wilk test. Continuous variables were expressed as mean ± standard deviation or median (minimum–maximum), while categorical variables were presented as n (%). The Mann–Whitney U test was used to compare groups based on the results of the normality test. Categorical variables were analyzed using the Chi-square test. Statistical analyses were performed using SPSS (IBM Corp. Released 2012. IBM SPSS Statistics for Windows, Version 21.0. Armonk: IBM Corp.). A Type I error level of 5% was considered statistically significant.

## 3. Results

**Figure 1.**
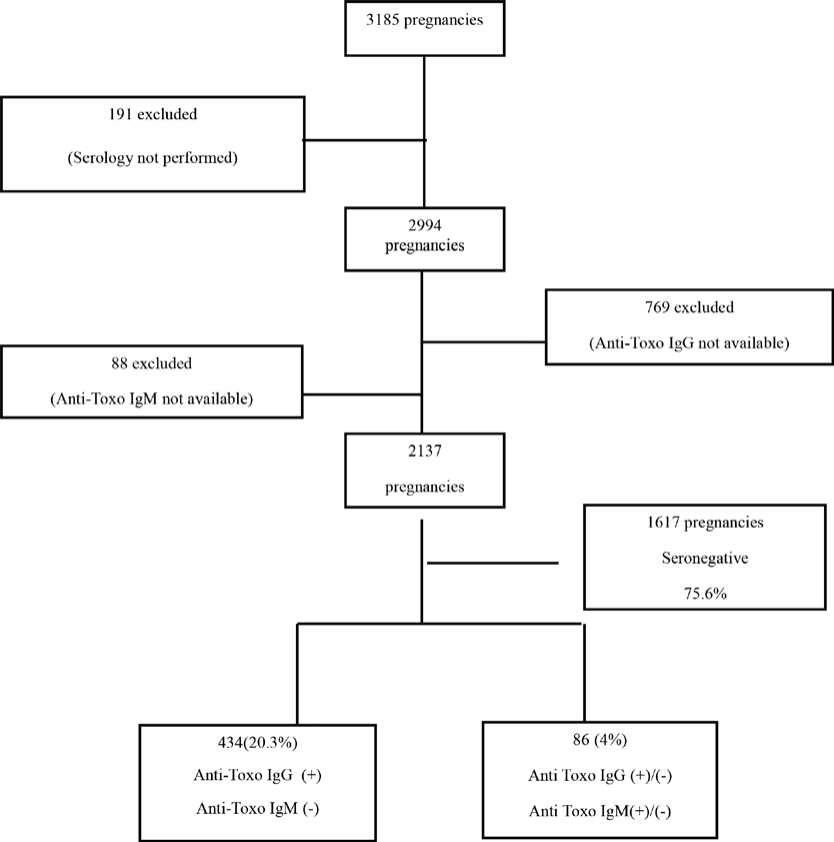
Toxoplasma serology data of pregnant women attending our center between 2015 and 2020.

Data from 3185 pregnant women between 2015 and 2020 were retrieved from the Hospital Information Management System. *T. gondii* serological testing was not performed in 191 cases, and these were excluded from the study. Among the remaining 2994 women, Toxo-IgG testing was not conducted in 769, and Toxo-IgM testing was not performed in 88 women. Consequently, 857 women were excluded due to the absence of either IgM or IgG testing, and the analysis was carried out with the remaining 2137 women (Fig. 1).

The average age of the participants was 29.8 years (range: 16–47). The median gravida was 2.0 (range: 1–15), the median parity was 1 (range: 0–9), and the median number of live births was 1 (range: 0–9). Educational status and residental area of patients were listed in Table 1. None of the pregnant women were immunocompromised due to other medical conditions or drugs. Only 3 of the IgM-positive women had cat in their house. Educational status, occupational status and residency did not differ significantly between all patient population and IgM-positive group (Table 1).

**Table 1:** Demographic characteristics.

	All patients	IgM positive patients	*P*-value
Age (year)	29.8 (16–47)*	28.3 (17–43)*	*P* = .655
Gravity	2.0 (1–15)*	2.07 (1–69)*	*P* = .87
Parity	1.0 (0–9)*	0.8 (0–2)*	*P* = .88
Educational status			
University	406 (18.9 %)**	14 (22%)**	*P* = .54
High school or middle school	1546 (72.3 %)**	44 (70%)**	*P* = .69
No education	185 (8.6 %)**	5 (8%)**	*P* = .84
Residence			
Rural	1389 (65%)**	42 (66.6%)**	*P* = .78
Urban	748 (35%)	21 (33.3%)	*P* = .89
Occupation			
Housewife	1142 (53.4%)**	26 (41.2%)**	*P* = .056
Workplace employee (teacher, nurse, accountant, lawyer…)	895 (42%)**	29 (46%)**	*P* = .51
Farmer	100 (4.6%)**	8 (1.2%)**	*P* = .005

* Mean (min–max).

* Value (percentage) *P* < .05 considered significant.

Of the 2137 pregnant women included in the study, initial Toxoplasma serological screening (*Toxo-IgM1* and *Toxo-IgG1*) revealed that 1617 women (75.6%) tested negative for both antibodies (seronegative). Toxo-IgG1 positivity with negative Toxo-IgM1 was observed in 434 women (20.3%), while 54 women (2.5%) had positive Toxo-IgM1 results (Table 2).

**Table 2:** Distribution of Toxo-IgM and Toxo-IgG positive and borderline pregnancies by trimester.

Serology	First Trimester	Second Trimester	Third Trimester	Total Patients
Toxo IgM1 (+)	44	13	1	58
Toxo IgM1 (borderline)	10	1	0	11
Toxo IgG1 (+)	388	99	9	496
Toxo IgG1 (borderline)	18	6	0	24
Toxo IgM2 (+)	20	11	0	31
Toxo IgM2 (borderline)	6	1	0	7
Toxo IgG2 (+)	42	16	1	59
Toxo IgG2 (borderline)	0	0	0	0

Toxo-IgM 1: initial value at first visit. Toxo-IgG 1: initial value at first visit. Toxo-IgM 2: follow-up value in positive/borderline cases. Toxo-IgG 2: follow-up value in positive/borderline cases.

In total, 69 women demonstrated either borderline or positive Toxo-IgM1 finding (3.2%). Of these, 5 were lost to follow-up, 4 underwent elective pregnancy termination, and 1 woman with confirmed acute toxoplasmosis experienced a spontaneous abortion at 7 weeks of gestation. This patient was excluded from polymerase chain reaction (PCR) and neonatal outcome analyses.

Follow-up serological assessments (*Toxo-IgM2, Toxo- IgG2*, and Ig G avidity) were conducted in 63 of the 69 patients. Among these, 35 (55.5%) remained IgM-positive, 7 (11.1%) had borderline IgM levels, and 21 (33.3%) converted to IgM-negative status.

**Figure 2.**
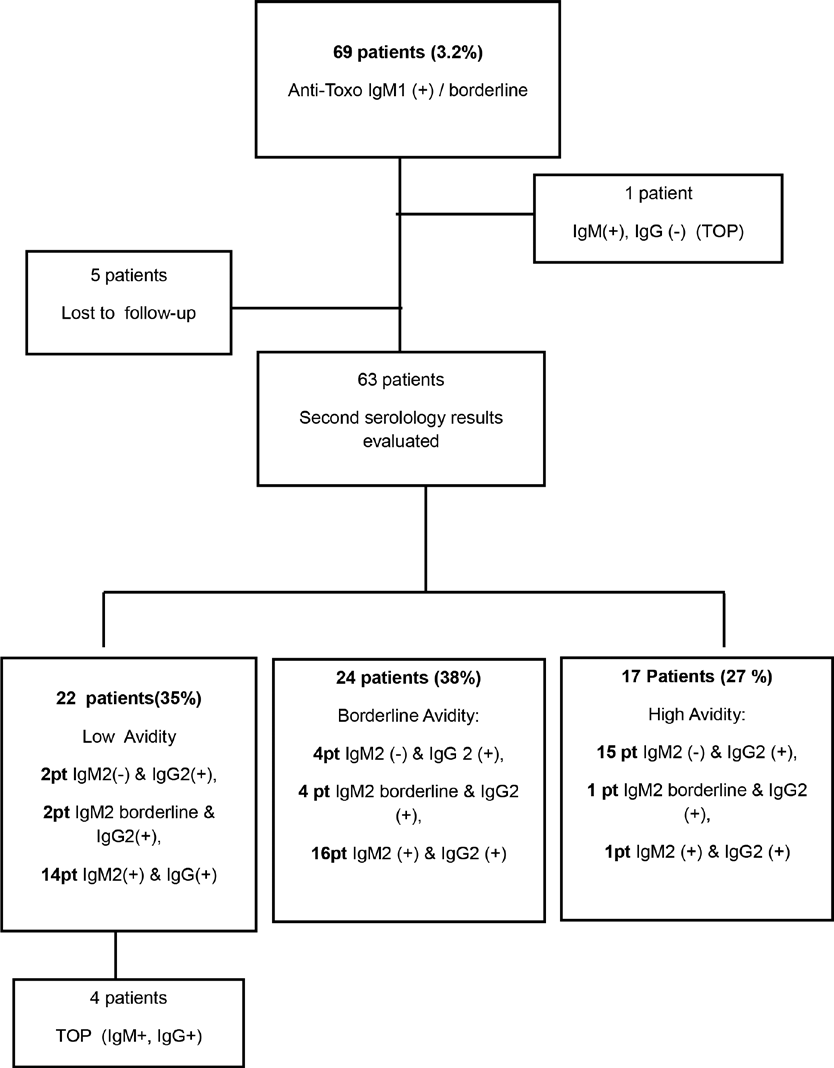
Follow-up Toxo-IgM2, IgG2, and IgG avidity2 results in women with positive or borderline Toxo-IgM1. TOP = termination of pregnancy.

IgG avidity testing revealed low avidity in 22 patients (35%), borderline in 24 (38%), and high avidity in 17 (27%) (Fig. 2). Four women opted for pregnancy termination following confirmation of acute toxoplasmosis (Fig. 2).

At the initial presentation, 127 patients (4.2%) with positive IgG levels underwent IgG avidity testing. Among them, 16 (12.5%) exhibited low avidity, 23 (18.1%) borderline avidity, and 88 (69.3%) high avidity. The mean gestational age at the time of testing was 8.57 weeks (range: 5–13) for the low avidity group, 8.8 weeks (range: 6–14) for borderline, and 8.9 weeks (range: 3–14) for high avidity cases.

In patients with suspected acute toxoplasmosis (low or borderline avidity) (46 patients – 2.1%), amniocentesis was recommended. Spiramycin treatment was started at a dose of 1 g (3 million UI) orally every 8 hours for all those patients. The procedure was accepted by 14 women (31%) and declined by 30 (69%) patients. The mean gestational age at the time of amniocentesis was 18.3 weeks. All 14 amniotic fluid samples tested negative for *T. gondii* by PCR (Table 3).

**Table 3:** Perinatal outcomes of Toxo-Igm-positive patients.

	Number of Patients	Gestational week
Spiramycine initiation	46 (2.1%)	14.3
Termination	4	12.5
Amniocentesis performed	14 (31 %)	18.3
Second trimester USG abnormality	1 (terminated)	21 (19–23)
Third trimester USG abnormality	0	30 (29–32)
Transfontanelle USG abnormality	0/38	4 wk postpartum
Neonatal serology		
IgM poz	0	Within 2 d and 2 wk postpartum
IgG poz	42	

USG = ultrasonography.

Among the 4 patients who underwent pregnancy termination, only 1 fetus exhibited multiple anomalies on obstetric ultrasonography and was terminated at 19 weeks. Although mother was prescribed she did not use spiramycin. The remaining 59 patients out of 63 patients were monitored through targeted second-trimester ultrasonography at 19 to 23 weeks, followed by additional fetal assessments. No sonographic signs indicative of congenital toxoplasmosis were identified except one (Table 3).

Of the total study population, 1284 women delivered at the study center. Among these births, 680 were male, 602 were female, and 2 newborns had ambiguous genitalia. The mean birth weight was 3082 grams in the seronegative group and 3201 grams in the seropositive group, with no statistically significant difference observed.

Among the 46 cases with confirmed or suspected acute toxoplasmosis, 4 were terminated and 38 women delivered at the study center. For the remaining 4 patients, neonatal data were obtained through follow-up telephone interviews with parents and from nationwide neonatal surveillance system records. Transfontanel ultrasonography results were available for 38 neonates (in those born at the institution), while postnatal serology was available for 42. All 38 ultrasonography findings were within normal limits. Toxoplasma IgM was negative in all 42 neonates, whereas IgG was positive in each case (Table 3).

## 4. Discussion

Although toxoplasmosis is a prevalent infection globally routine screening during pregnancy is implemented in only a limited number of countries. Congenital toxoplasmosis is frequently asymptomatic; however, it can result in serious complications such as chorioretinitis, hydrops fetalis, intrauterine fetal demise, and neurological impairments. Maternal infection is typically asymptomatic, but the risk of fetal transmission increases with each advancing trimester.^[1,2,7]^ Consequently, several countries recommend routine serological screening during the first trimester. In France, where such screening has been mandatory since 1992, coupled with improved hygiene standards, the seroprevalence among pregnant women has declined from 80% to approximately 30%, with an acute infection rate during pregnancy of 0.2% to 0.25%.^[2,10]^

In Turkey, reported IgG toxoplasmosis seropositivity rates among pregnant women range from 28.8% to 57%.^[11–13]^ In our study, the observed IgG seropositivity rate was 22%. This rate is similar with rate 28.8% in women presented in literature from our city and in Turkey.^[12,13]^ The similar rates may be due to the fact that communities across different parts of the country share comparable sociocultural and socioeconomic characteristics. A 2020 global survey on women of reproductive age reported an average toxoplasmosis seroprevalence of 27.2%, while the rate in Turkey was 34.7%.^[12]^

The decreasing incidence and prevalence of *Toxoplasma* infection in Europe, along with the limited availability of data regarding the incidence of toxoplasmosis during pregnancy,^[14]^ have led to a fragmented epidemiological understanding and renewed debate about the necessity of routine serological screening in pregnancy. Given that our country occupies a key geographic position linking Asia, Europe, and Africa – and hosts a large refugee population with a high reported seroprevalence rate (64%)^[13]^ – there is a strong rationale for establishing a national screening program.

A positive IgM result does not definitively indicate a recent infection acquired during pregnancy. False-positive IgM findings or persistent IgM positivity – observed in up to 27% of chronically infected individuals – require careful interpretation.^[14]^ Therefore, simultaneous assessment of IgG levels, IgG avidity testing, and serial serological follow-up are crucial for accurate diagnosis. IgG avidity testing is widely used to differentiate between acute and chronic infection.^[8–10]^

In our cohort, 69 women (3.2%) tested IgM-positive during the initial evaluation. Following confirmatory testing with IgG and IgG avidity assays, and a second serological assessment, only 22 cases (1.02%) were confirmed to have acute toxoplasmosis, other 24 were followed as suspected cases according to IgG avidity. Without this additional evaluation, all these 63 women might have been unnecessarily counseled toward pregnancy termination or invasive procedures. The rate of IgM seropositivity in our city was found in women was 2.02% which is similar to our result.^[12]^ The prevalence of acute Toxoplasmosis is a bit higher than the 3-year average incidence of 0.192% reported by an Italian study group which may be consequence of geographical difference.^[14]^

A high-avidity IgG result suggests that the infection occurred at least 3 to 5 months earlier.^[7,15]^ During pregnancy, IgG avidity testing plays a crucial role in distinguishing acute from chronic infection, thereby guiding appropriate management.^[14]^ Even when acute infection is excluded based solely on IgM and IgG results, maternal-fetal transmission has been reported in 2.6% of cases. Inclusion of IgG avidity testing reduces this rate to 0.2%.^[7]^ The timing of serological testing is also critical. The first trimester is considered ideal, as IgG avidity testing can provide retrospective information for up to 16 weeks. In our cohort, we were able to adhere to this recommendation, with a mean testing time of 8.5 weeks of gestation.

Lappalainen et al assessed anti–*T. gondii* IgG avidity in 44,181 serum samples from 16,733 pregnant women across trimesters, identifying low avidity with IgM positivity in 42 cases. Of these, congenital toxoplasmosis developed in 4 newborns.^[7]^ Approximately 10% of infants born to mothers with acute infection and low avidity developed congenital toxoplasmosis, whereas none of the children of women with high avidity IgG in the first trimester showed evidence of infection during one-year follow-up.^[5]^ High avidity results in 17 women (27%) during the first trimester precluded unnecessary treatment or intensive follow-up. In contrast, patients with low or borderline avidity values were treated with spiramycin in the early weeks of gestation, even in the absence of maternal symptoms or abnormal fetal ultrasonographic findings.

In France, prenatal treatment reduced the occurrence of ocular sequelae in infants born to seropositive mothers to 30%, compared to 70% in the United States, where no screening is routinely performed.^[9,10]^ Several studies support that antenatal screening and early treatment reduce vertical transmission and improve neonatal outcomes.^[2,8,15,16]^ In this context, early pregnancy serological evaluation in Turkey could facilitate timely diagnosis and treatment, potentially improving neonatal outcomes.

Despite its utility, the IgG avidity test should not be used in isolation, as low or borderline results may yield ambiguous interpretations.^[7,8,15]^ In such cases, detection of *T. gondii* DNA by PCR in amniotic fluid offers a more definitive diagnosis and allows for treatment optimization.^[15]^

A key limitation of our study was the relatively low acceptance rate for amniocentesis. Unlike studies such as Vimercati et al., which included PCR and neonatal serology data, some women in our study delivered at external centers, limiting access to neonatal follow-up data^[15]^

In Turkey, preconception visits are uncommon. As prepregnancy serological data were unavailable, none of the seropositive pregnant women in our cohort could be classified as having a confirmed infection according to the modified Lebech classification. Thus, our “probable infection” group may include some cases of confirmed infection. Furthermore, all 4 women with confirmed diagnoses opted for pregnancy termination, precluding assessment of fetal transmission.

The SYROCOT meta-analysis reported no significant difference in transmission rates between spiramycin and the combination of pyrimethamine–sulfadiazine.^[5]^ However, in a 2018 prospective controlled study by Mandelbrot et al., PCR positivity was twice as high in the spiramycin-treated group, although this difference did not reach statistical significance.^[5,17]^

Spiramycin is most effective when initiated within 8 weeks of maternal seroconversion. Even if PCR results are negative and fetal imaging is normal, spiramycin should be continued until delivery.^[17]^ Spiramycin is not associated with teratogenicity and is used to reduce transplacental transmission.^[6]^ In another study, among 61 women diagnosed during the first trimester, 55 received spiramycin and had no PCR positivity, whereas 4 of 6 untreated women had positive amniotic fluid PCR results.^[18]^ In our study, none of the patients who received early spiramycin treatment demonstrated PCR positivity for *T. gondii* in amniotic fluid. Furthermore, none of the corresponding neonates showed clinical signs or laboratory evidence of *T. gondii* infection.

International approaches to *T. gondii* screening during pregnancy vary significantly. Routine screening is mandated in several European countries, including France, Austria, Belgium, and Italy.^[2,10,14,16]^ In contrast, countries such as the United States and the United Kingdom recommend targeted screening primarily for pregnant women presenting with abnormal ultrasound findings.^[9,16]^ In France, seronegative women undergo repeated serological testing throughout pregnancy until delivery. However, major health organizations in the US, including the American College of Obstetricians and Gynecologists and the Centers for Disease Control and Prevention (CDC), do not endorse universal screening; rather, they emphasize preventive measures and patient education.^[9]^ Management of congenital toxoplasmosis in France has been reported to result in minimal impact on the quality of life and visual function among affected adults.^[19]^ Conversely, literature from the US, where comprehensive antenatal screening programs are lacking, indicates that infected children tend to experience more severe disease manifestations and poorer clinical outcomes.^[19]^ Several factors, including parasite and host genetics as well as infecting strains, may contribute to these differences in clinical scenarios observed across continents. The availability of antenatal treatment in France, contrasted with its absence in the US, also likely plays a significant role. Congenital toxoplasmosis is estimated to affect about 1/10 000 live births in the United Kingdom. No studies were identified that addressed the uncertainty around the burden of disease in either congenital or maternal toxoplasmosis infections, within a UK population. The UK has, like other countries, comprehensive literature offering prenatal advice on prevention of toxoplasmosis. However a recent Cochrane review suggested that this information increases awareness but, crucially, not preventive behavior. This review and poor understanding of the source of infection (uncooked meats, the environment and cats) questions the assumption that information giving is effective in primary prevention.^[20]^ The severity of congenital toxoplasmosis has been shown to decrease with timely suspicion, diagnosis, and treatment. Similarly, in Turkey, neither the Ministry of Health nor the Turkish Perinatology Society advocate for routine toxoplasmosis screening during pregnancy.^[13,21]^

## 5. Conclusion

Of the 2137 pregnant women included in the study, only 69 (3.2%) were found to be IgM-positive at initial serological screening. Among these, 22 were diagnosed with acute toxoplasmosis based on repeat serological testing and IgG avidity assays performed at a 2-week interval. A total of 46 women received spiramycin treatment for confirmed or suspected acute toxoplasmosis. Acceptance rate of invasive procedure is low (30%) but amniotic fluid PCR results revealed no evidence of fetal infection in treated cases. Additionally, none of the neonates exposed to spiramycin exhibited abnormalities on transfontanelle ultrasonography.

Prospective studies with larger cohorts are warranted to validate these findings and further inform screening strategies.

## Author contributions

**Conceptualization:** Bilge Cetinkaya Demir, Hilal Ozkan.

**Data curation:** Bilge Cetinkaya Demir, Oguzhan Yuruk, Yasemin Heper.

**Formal analysis:** Yasemin Heper.

**Investigation:** Bilge Cetinkaya Demir, Oguzhan Yuruk, Hilal Ozkan.

**Methodology:** Bilge Cetinkaya Demir, Hilal Ozkan.

**Project administration:** Oguzhan Yuruk.

**Resources:** Yasemin Heper.

**Software:** Oguzhan Yuruk.

**Supervision:** Bilge Cetinkaya Demir, Hilal Ozkan.

**Visualization:** Yasemin Heper.

**Writing – original draft:** Bilge Cetinkaya Demir.

**Writing – review & editing:** Bilge Cetinkaya Demir.
